# Energy Harvesting from Ankle Flexion During Gait Using Flexible CdS and PVDF Sensors

**DOI:** 10.3390/mi16060698

**Published:** 2025-06-11

**Authors:** Kimberly Trevizo, Luis Santana, Manuel Chairez, Amanda Carrillo, Rafael Gonzalez-Landaeta

**Affiliations:** 1 BIOCIM Research Group, Engineering and Technology Institute, Autonomous University of Ciudad Juárez (UACJ), Ciudad Juárez, Chihuahua 32310, Mexico; al251251@alumnos.uacj.mx; 2 ByNEF Research Group, Engineering and Technology Institute, Autonomous University of Ciudad Juárez (UACJ), Ciudad Juárez, Chihuahua 32310, Mexico; al216560@alumnos.uacj.mx (L.S.); manuel.chairez@uacj.mx (M.C.); amanda.carrillo@uacj.mx (A.C.)

**Keywords:** cadmium sulfide, dorsiflexion, energy harvesting, gait, piezoelectric films, polyvinylidene fluoride

## Abstract

In this work, energy was harvested from ankle flexion during gait. For this, two piezoelectric thin films were tested: PVDF and CdS. The PVDF film was a commercial option, and the CdS film was fabricated in our laboratory. Deposition of the CdS film is also reported in this work. Energy harvested during gait from heel strike and ankle flexion was compared. Tests were performed with 10 healthy volunteers walking on a treadmill at 1.2–1.5 km/h. The volunteers wore a sock with piezoelectric films incorporated in the heel and ankle joint (talocrural joint). Tests were performed first with the PVDF film and then with the CdS film. The CdS thin film obtained a *d*_33_ coefficient of 1.4928 nm/V, indicating high electrical energy generated from strain-stress. The talocrural joint generated the most energy: 11.359 μJ for the PVDF film and 0.854 μJ for the CdS film. Although the CdS film generated less energy than the commercial option, it was shown that harvesting energy from ankle flexion increased the energy harvested by more than 700% during gait compared to the energy harvested from heel-to-ground impact.

## 1. Introduction

In biomechanical energy harvesting, especially in the podiatric area, piezoelectric sensors have been used to capture joint and limb movements. Among the most used sensors are PVDF (polyvinylidene fluoride), which are recommended in applications with high amplitude and low frequency movements [1]. Currently, piezoelectric sensors have been structured in thin films of piezoelectric material. There are different classes of materials used for energy harvesting, such as ferroelectric, piezoceramic, non-ferroelectric, piezopolymeric, and composite materials. Some studies indicate that PZT (lead zirconate titanate) sensors face challenges in being incorporated into flexible devices due to their rigidity [2,3,4]; on the other hand, PVDF, being more flexible, is more used in wearable devices.

Over the years, different energy harvesting systems have been implemented using wearable devices for daily use. To mention a few, there are backpacks, shoes, watches, belts, etc. [5,6,7]. In the podiatric area, the pressure exerted by an individual’s full weight during walking is used to generate energy with piezoelectric, triboelectric, electrostatic, and electromagnetic sensors [8], where better results have been obtained regarding current, voltage, and power [9].

In some studies proposed so far, PZT sensors have been the most implemented sensors for applications where the energy of the impact between the heel and the ground during walking is harvested. Over the years, various positions, attachments, and types of footwear, such as heels, have been studied to focus the forces applied to the sensor, producing very favorable results, especially in shoes that have a narrow heel area and higher elevation [10]. On the other hand, energy harvesting through piezoelectric sensors is one of the most effective methods, offering numerous opportunities for research, improvement, innovation, and, above all, efficiency and sustainability.

There are still areas of the body that have not been explored for biomechanical energy harvesting during daily life activities [10]. PVDF sensors within the podiatric area have only been implemented in the plantar area of the foot as an impact harvester. The problem with harvesting energy from heel strike is that the energy is only harvested during the stance phase of gait. The talocrural joint, which generates biomechanical energy during all phases of walking, has not yet been explored; only the elbow and knee joints have been studied, which produce a significant amount of biomechanical energy during walking [11].

Various semi-crystalline polymeric materials, such as PVDF, contain very low piezoelectric coefficients compared to PZT due to the semiconductor materials from which they are made. Although polymeric materials offer significant advantages, such as biocompatibility, a deficiency in the piezoelectric coefficient still exists, which limits their potential integration into wearable systems where substantial amounts of energy harvesting are desired [12]. Currently, various studies have enhanced the piezoelectric coefficient of PVDF sensors by introducing different types of semiconductors through techniques such as electrospinning. One of the options is cadmium sulfide (CdS), although its benefits remain unclear. Different semiconductor materials have been compared, and it was found that adding CdS nanoparticles to CdS films and subjecting them to annealing between each added layer improves the level of voltage generated [13,14]. CdS is a typical n-type semiconductor, characterized by its yellow color. It can also be classified as a binary compound due to the combination of Cd and S, and as a chalcogenide because it involves a bond between a metal and a non-metal from group 6A of the periodic table. CdS can exhibit cubic and hexagonal crystalline structures, with the latter being wurtzite. This structure demonstrates piezoelectric behavior, showing a typical *d*_33_ coefficient of 10.32 nC/N. This property enables the material to be used in various energy harvesting applications [15]. For example, in the human body, CdS NPs have been explored in various studies in the fields of biosensors, bioimaging, and other biomedical applications. Favorable results have been obtained, considering it one of the most promising chalcogenide materials for use in the biomedical field [16].

In this work, two points of interest have been addressed: enhancing the energy harvested during walking by exploiting the ankle flexion and comparing PVDF piezoelectric films and CdS films doped with CdS nanoparticles in energy-harvesting applications. Comparison of these sensors will be conducted by placing them at the talocrural joint (the junction between the ankle and the rest of the foot) and the plantar region.

The paper is organized as follows. Section 2 briefly describes the kinematics of the ankle during one cycle of gait. Section 3 describes the film deposition, characterization, and energy-harvesting circuits and methods. Section 4 discloses the experimental results and the discussion, and Section 5 draws the main conclusions.

## 2. Kinematics of the Ankle

**Figure 1:**
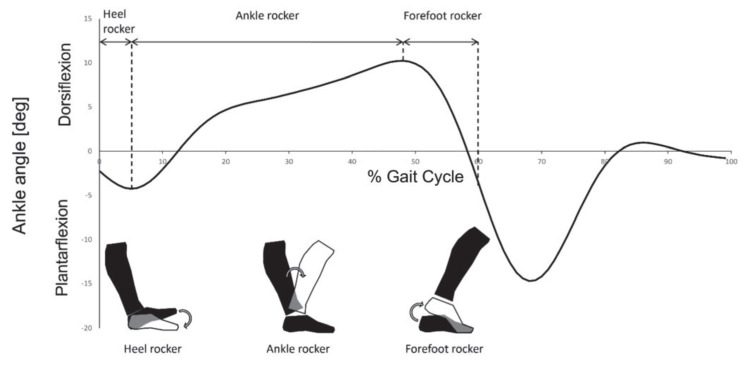
Graphical representation of dorsiflexion and plantar flexion during one cycle of gait [17].

The gait of a subject consists of a series of cyclical movements. These movements involve different parts of the lower extremities, from the hips to the feet, generating biomechanical energy. The gait cycle has two main phases: swing and stance. The swing phase accounts for 40% of the gait cycle, while the support phase accounts for 60%. Heel strike occurs during less than 30% of the gait cycle. However, ankle flexion (dorsiflexion and plantarflexion) takes place during almost 80% of the gait cycle (Figure 1), as it involves both phases. In this work, we consider that placing a piezoelectric film on the talocrural junction allows for harvesting of biomechanical energy during ankle dorsiflexion and plantarflexion. Thus, a greater portion of the gait cycle can be harvested compared to heel strike. Regarding the energy generated by the piezoelectric harvesters, if we assume that heel strike generates a narrow pulse-type signal of duration *τ*, and that ankle dorsiflexion and plantarflexion generate a sinusoidal signal of duration *T*, the energy for each case would be

(1) $$E_{\mathrm{HS}}=V_{HS}^{2}\tau \;(\mathrm{Joules})$$

(2) $$E_{\mathrm{FLEX}}=V_{FLEX}^{2}\frac{T}{2}\;(\mathrm{Joules})$$

where $V_{\mathrm{HS}}$ and $V_{\mathrm{FLEX}}$ represent the heel strike amplitude and ankle flexion amplitude, respectively. From Equations (1) and (2), assuming $V_{\mathrm{HS}}=V_{\mathrm{FLEX}},$ if τ≪T, then $E_{\mathrm{HS}}\ll E_{\mathrm{FLEX}}.$

## 3. Materials and Methods

### 3.1. Reagents for CdS Thin Film Deposition

The substrates used to deposit the materials were soda lime glass (Type II, ASTM E438-75 mm × 25 mm × 0.9 mm, CORNING, Tewksbury, MA, USA) and indium tin oxide-coated PET (ITO/PET, surface resistivity of 100 Ω/sq, 25.4 mm × 25.4 mm × 7 mm sheet, Sigma Aldrich, St. Louis, MO, USA).

The solvents used for substrate cleaning were acetone (ACS, 99.5%, Monterrey, Mexico), isopropyl alcohol (CTIR UPS, 99.986%, Monterrey, Mexico), and deionized water (Ciudad Juarez, Mexico). The reagents used for CdS thin film deposition by chemical bath were cadmium chloride (CdCl_2_, 99%, Fermont, NJ, USA), sodium citrate (Na_3_C_6_H_5_O_2_, 99.9%, Fermont, Monterrey, Mexico), potassium hydroxide (KOH, 87.3%, Fermont, Monterrey, Mexico), borate buffer with a pH of 10 (J.T. Baker, Estado de Mexico, Mexico), thiourea (CH_4_N_2_S, 99.4%, J.T. Baker, Ecatepec, Edomex, Mexico), and methanol (CH_4_O, 99.92%, CTR, Monterrey, Mexico).

### 3.2. Chemical Bath Deposition of CdS Thin Films

The soda lime glass and ITO/PET substrates were cleaned in an ultrasonic bath. The substrates were covered with isopropanol and sonicated for 15 min (only the glass substrates were previously cleaned with acetone, and then the process was repeated with deionized water). After this, the substrates were dried.

A mixture of the CdS precursors was prepared by adding 31.5 mL of deionized water, 9 mL of CdCl_2_, 9 mL of sodium citrate, 3 mL of KOH, 3 mL of borate buffer with a pH of 10, and 4.5 mL of thiourea. For thin film deposition on substrates, the solution temperature was maintained at 43 °C ± 1 °C for 33 min [18]. Then, the CdS thin films deposited over the glass were cleaned in an ultrasonic bath for 10 min, and they were covered with methanol. This procedure was carried out two additional times to apply three layers of CdS thin films, stacking one on top of another. This same process was used to deposit the material on the ITO/PET substrates, replacing the glass substrates.

### 3.3. Characterization Methods for CdS Thin Films

Optical characterization was carried out using a double-beam ultraviolet-visible spectrophotometer (6850 Jenway, from 300 to 1100 nm, Dunmow, UK). Microstructural characterization was performed by scanning electron microscopy (JEOL JSM-7000 F, with 10,000X to 30,000X, Tachikawa-shi, Japan). The crystalline structure was studied with X-ray diffraction (Xpert Pro Panalytical D42877, operated at 40 kV and 30 mA, λ = 1.54 Å, scan velocity = 2θ at 0.5°/min). The electrical properties were analyzed with a digital multimeter (KEYSIGHT 344S04, 1.00000 VAC, Santa Rosa, CA, USA) and with an impedance analyzer (KEYSIGHT E4990A, from 20 Hz to 100 kHz, Santa Rosa, CA, USA). The piezoelectric properties were measured by atomic force microscopy (Asylum Research MFP-3D Infinity, scan velocity of 0.8 Hz, silicon AFM probes of 160 μm and contact resonance frequency of 1100 kHz, Abingdon, UK).

### 3.4. Dynamic Characterization of Sensors

The length of the talocrural joint was estimated to be approximately 70–80 mm, leading to the selection of a PVDF sensor with similar dimensions. So, the LDT2-028K (TE Connectivity, Denver, CO, USA) was chosen (L × W × t: 73 mm × 16 mm × 28 μm), with a capacitance of 2.85 nF. The dimensions of the CdS film developed by the ByNEF research group were 25 mm (width) × 75 mm (length) × 110 nm (thickness). The capacitance was estimated by electrical impedance spectroscopy.

**Figure 2:**
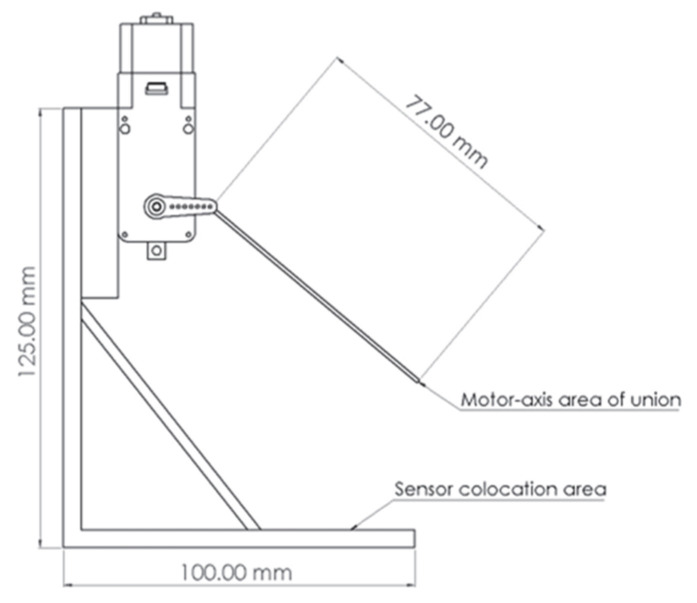
PLA base designed to dynamically characterize PVDF and CdS films.

Both sensors were mounted on a PLA base designed and fabricated using a SV01 PRO 3D printer (Sovol, Shenzhen, China). This base ensured proper sensor positioning and integration with a DC motor-reduction system (Figure 2). The setup induced sinusoidal deformations in the sensors at frequencies ranging from 1.5 Hz to 3.5 Hz, mimicking real-world walking conditions; that is, low frequencies and high-amplitude movements. A controller (L293D) and an Arduino Uno board regulated the motor speed with a 3.3 V and 5 V power supply.

**Figure 3:**
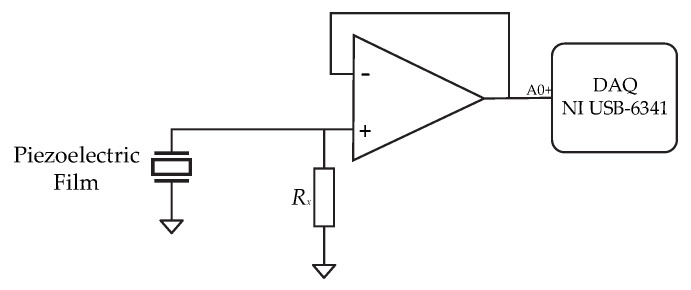
Impedance-coupling circuit.

Each sensor was connected to an impedance-coupling circuit to reduce loading errors (Figure 3). The output voltage was measured using a data acquisition system, model NI USB-6341. A frequency sweep was conducted at 1.5 Hz, 2.5 Hz, and 3.5 Hz. The resistance *R_x_* varied from 1 MΩ to 100 MΩ to estimate the maximum power that can be harvested using the test conditions. The theoretical optimal resistance for maximum power transfer was estimated using Equation (3), considering sensor capacitance $C_{S}$.

(3) $$R_{x}=\frac{1}{jwC_{S}}$$

The power *P* for each resistance was calculated using Equation (4), where $V_{rms}$ represents the root mean square voltage across $R_{x}$.

(4) $$P=\frac{V_{rms}^{2}}{R_{x}}$$

### 3.5. Energy-Harvesting System

**Figure 4:**
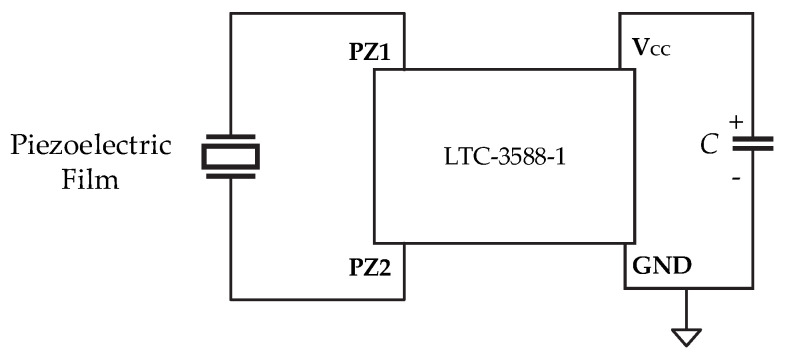
Setup for measuring the charging voltage of the capacitor when the sensors were deformed, using the system shown in Figure 1.

The sensor output was connected to a Nanopower Energy Harvesting circuit LTC-3588-1 (Analog Devices, Norwood, MA, USA), which included a rectifier bridge. At the output of the rectifier bridge, a 68 μF/16 V electrolytic capacitor was connected (Figure 4), and its voltage was monitored using an NI USB-6341-BNC acquisition system (National Instruments, Austin, TX, USA).

### 3.6. Measurement Protocol

**Figure 5:**
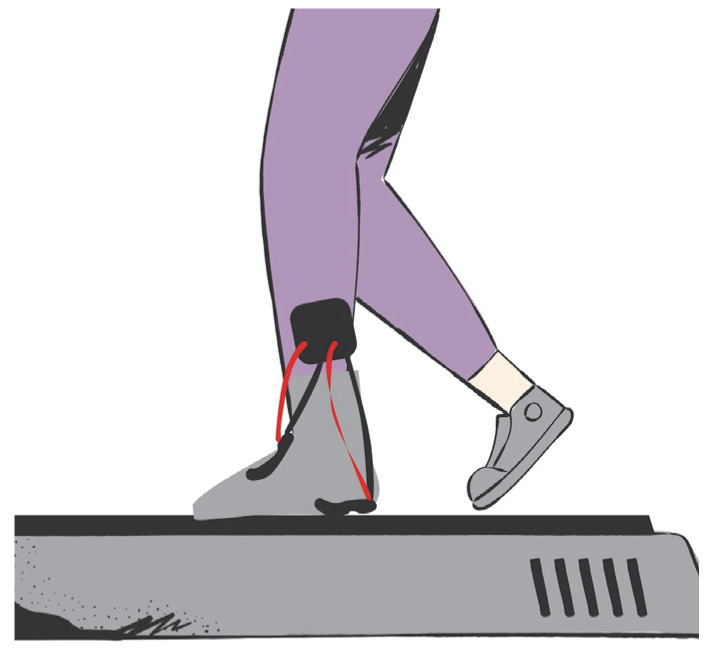
Locations of piezoelectric sensors for energy harvesting during walking.

The study involved 10 healthy volunteers, three females and seven males, with the following characteristics (mean ± SD): age = 24 ± 2 years, height = 1.75 ± 0.09 m, and weight = 76.2 ± 7.1 kg. The exclusion criteria were: suffering from any heart disease, having hypertension, being pregnant, or being a minor. The measurement protocol was approved by the Ethics Committee of the UACJ (CEI-2024-1-1154). The protocol consisted of walking on a WESLO CADENCE DS 10 treadmill for five minutes at 1.2–1.5 km/h. During the tests, one piezoelectric sensor was placed at the talocrural joint, and one piezoelectric sensor was placed at the heel of the preferred foot (Figure 5). Each volunteer performed the test twice: once using PVDF films and once using CdS films.

**Figure 6:**
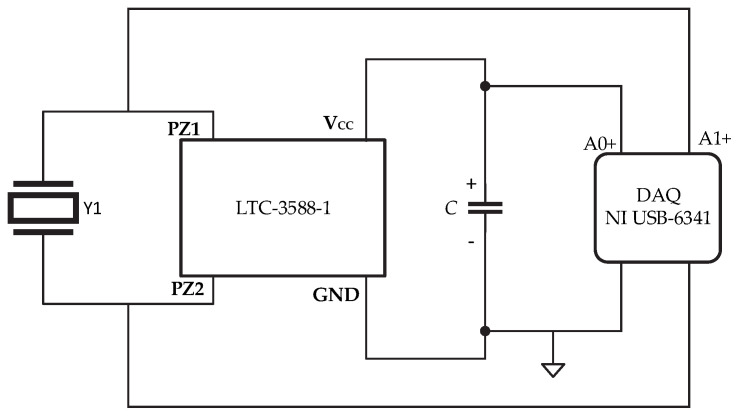
Energy-harvesting circuit.

Data acquisition was conducted using the circuit shown in Figure 6, where the waveform of the output voltage of the piezoelectric film and the voltage of the capacitor were simultaneously measured using the NI USB-6341 connected to a laptop operating on battery power to prevent electrical interference.

#### 3.6.1. Sensor Positioning During Walking

**Figure 7:**
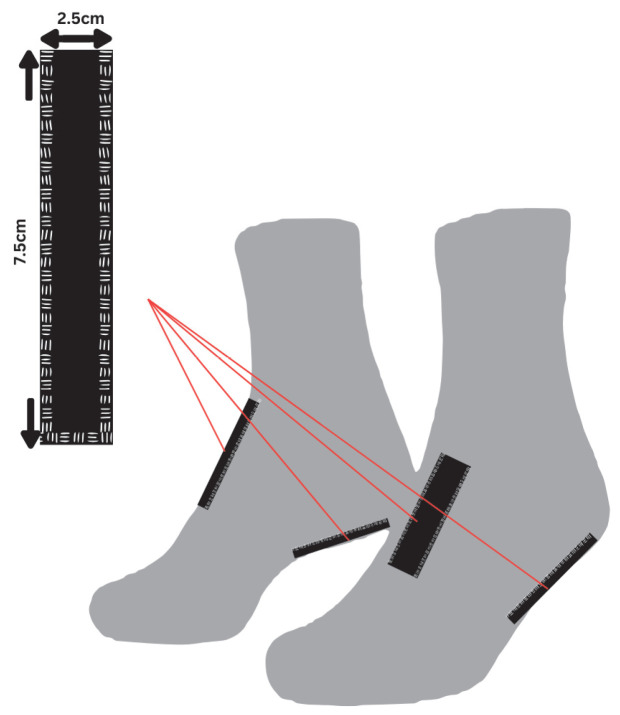
Sensor pocket positioning diagram.

A standard cotton sock was modified to include two compartments for sensor placement at the talocrural and heel regions, as shown in Figure 7. Compartments were sewn to accommodate the sensor dimensions (~75 mm × 25 mm). Three different sock sizes (S, M, and L) were prepared to fit the foot sizes of the volunteers.

#### 3.6.2. Energy-Harvesting Housing Design

**Figure 8:**
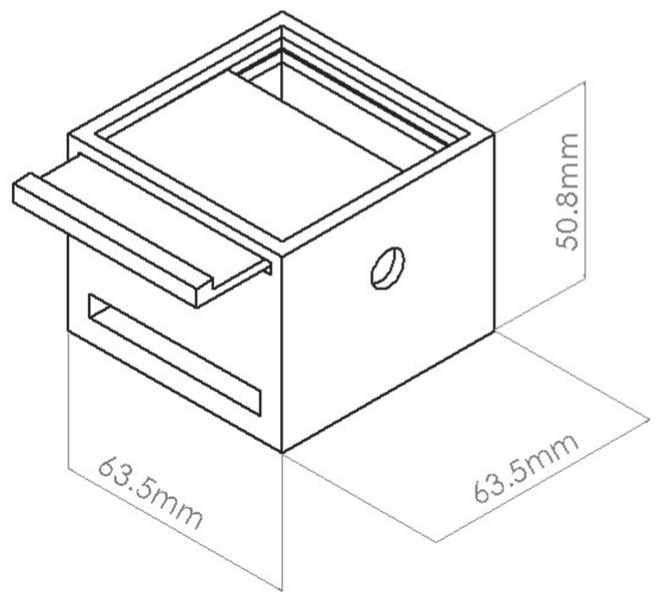
Housing for the energy-harvesting circuits.

To facilitate energy-harvesting analysis, a portable system was designed to be secured to the participant’s leg using an elastic strap design, as shown in Figure 8. The housing included a protoboard slot, cable entry/exit holes, and a shielding layer of aluminum tape to minimize interference.

The harvested energy (E) was estimated by Equation (5), where *C* is the capacitance of the storage capacitor and *V* is the charging voltage.

(5) $$E=\frac{1}{2}CV^{2}$$

## 4. Results and Discussion

**Figure 9:**
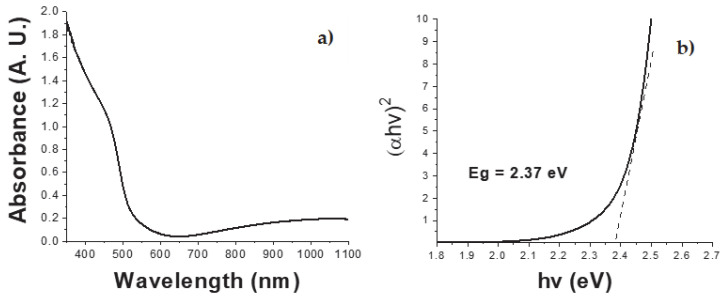
(**a**) UV-Vis absorbance spectra of CdS thin film (three layers). (**b**) Tauc variable versus energy plot for CdS thin film (three layers).

The absorbance spectrum of the CdS thin films deposited on a glass substrate is shown in Figure 9a. The films exhibit high absorbance in the UV region, indicating strong light absorption in this range. This behavior is typical of semiconductors, which display high UV absorption due to electronic transitions from the valence band to the conduction band [18]. Additionally, the films present an absorption edge at approximately 523 nm in the visible range, which aligns with reported values for CdS thin films in the literature [14].

The optical band gap was calculated using a Tauc plot (Figure 9b) and Equation (4), resulting in a band gap energy of 2.37 eV, which is within the expected range for CdS [14].

(6) $$ahv=A{\left(hv-Eg\right)}^{n}$$

where α, *hv*, *A*, and *Eg* correspond to the absorption coefficient, the incident photon energy (Planck constant and light vibration frequency), the proportionality constant, and the band gap energy, respectively. Finally, *n* is a constant with a value of 0.5, assuming a direct band gap.

**Figure 10:**
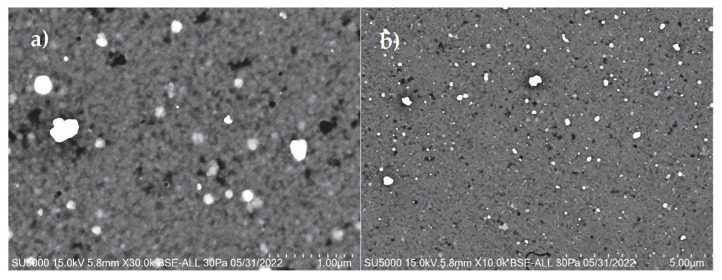
SEM micrographs of the surface of CdS thin film (three layers). (**a**) 30,000× magnification, (**b**) 10,000× magnification.

Figure 10 shows SEM images of the thin film at 10,000 and 30,000 magnifications. Homogeneous morphology is shown at the surface of the CdS film deposited via CBD. Nanocrystalline grains can be seen in a compact granular structure, with very well-defined grain boundaries; these are the characteristics of the ion-by-ion growth mechanism of the chemical bath deposition process. Additionally, clusters are visible in the micrographs. These aggregates are characteristic of this ion-by-ion deposition and appear as white dots distributed across the entire surface of the material. They form as a result of an increase in the concentration of semiconductor ions. This can be linked to the three layers deposited, which show an increase in the concentration of CdS and lead to the formation of clusters on the surface of the thin film [19]. Although the film has three layers of CdS without an annealing process, there is no evidence of cracks due to mechanical stress induced by the increment of layers, from two to three [14].

**Figure 11:**
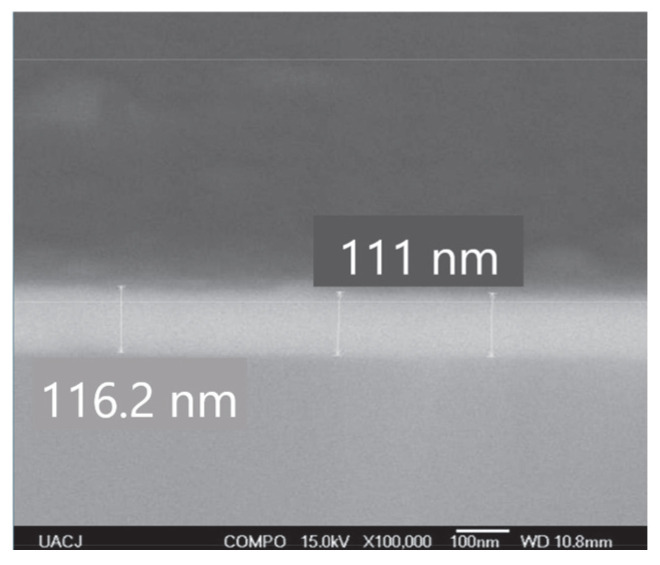
SEM micrographs of a cross-section of the CdS thin film (three layers).

There is an inverse relationship between film thickness and its capacitance. In this study, the CdS thin film measured approximately 111 nm in thickness, as shown in Figure 11, while the PVDF was 28 µm thick. However, despite CdS being thinner, it exhibited lower capacitance, which can be attributed to other material properties, including the electrical permittivity coefficient of CdS. Additionally, other aspects, such as the relationship between *d*_33_ and thickness, should be analyzed, as has already been studied for ceramic materials [20]. Furthermore, it is important to highlight that the efficiency of mechanical-to-electrical energy conversion is higher in thicker piezoelectric films. This is attributed to increased domain mobility, a reduction in the film interface, the effects of restraints, and residual tensile stresses.

**Figure 12:**
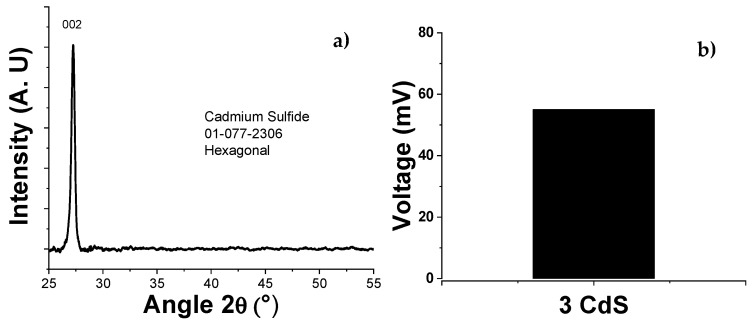
(**a**) XRD pattern for CdS thin film (three layers). (**b**) Electrical response of the CdS thin film (three layers) when subjected to mechanical stress by ultrasonic vibration with a frequency of 46 kHz.

Figure 12 shows the XRD patterns obtained from the CdS thin film. The peak located at 2θ = 27.2 indicates a preferential orientation along the (002) hexagonal plane [21]. The tetrahedral structure of the ions causes a non-centered crystal structure, meaning that, when not mechanically stressed, the centers of mass of the positive and negative ions coincide at the center of the tetrahedron, resulting in a lack of net polarization in the unit cell. However, when mechanical stress is applied at a vertex of the tetrahedron, the centers of mass of the ions shift, generating net polarization or charges on the crystal surface (piezoelectric effect) [22].

The films were subjected to ultrasonic vibrations of 46 kHz, which, through the piezoelectric effect, were able to convert mechanical energy into a potential difference. This vibration in the thin film causes deformation by bending the structure, generating voltage.

**Figure 13:**
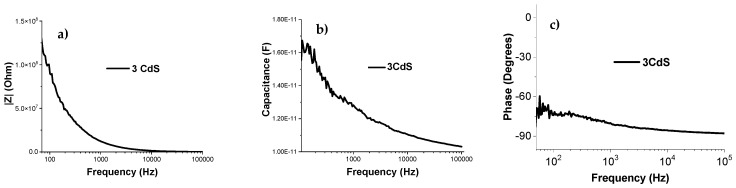
(**a**) Modulus of the electrical impedance of CdS thin film (three layers), (**b**) parallel capacitance of the electrical impedance of CdS thin film (three layers), and (**c**) phase angle of CdS thin film (three layers).

The magnitude of electrical impedance, phase angle, and parallel capacitance were measured in a frequency range from 20 Hz to 100 kHz. This range was chosen to avoid inductive effects from the connection cables at high frequencies. In Figure 13a, it can be observed that, at low frequencies, the film exhibits high impedance, and as the frequency increases, the impedance decreases. This behavior aligns with the expected response of a piezoelectric material, which has a significant capacitive component. At high frequencies, above 80 kHz, values in the range of hundreds of kΩ were obtained.

In Figure 13b, the film changes its capacitance with frequency, starting at approximately 16 pF at low frequencies and decreasing to 11 pF at high frequencies. This effect can be attributed to the lack of thermal treatment, which may result in a more disordered structure in the CdS layers [14]. This could result in more defects, trapped charges, and/or non-uniform interfaces, which can affect capacitance.

A phase angle close to −90 degrees (Figure 13c) indicates a predominantly capacitive response in the film. In a piezoelectric material, this behavior can be attributed to the capacitive nature associated with the material’s piezoelectric response, as seen in the XRD analysis. At the microscopic level, piezoelectric materials can have piezoelectric domains that polarize in response to mechanical stress. These domains can behave similarly to capacitors in a circuit, contributing to the capacitive component of the impedance. The persistence of a phase angle close to −90 degrees suggests a pronounced capacitive response, which is consistent with the piezoelectric properties of the material [23].

**Figure 14:**
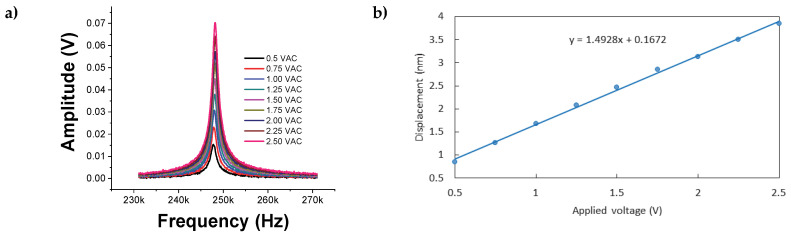
(**a**) Piezoresponse of the three-layer CdS film and (**b**) displacement vs. applied voltage.

The piezoelectric properties of the film were studied using PFM, as shown in Figure 14a. Using the piezoelectric calibration factor of the equipment (54.7 nm/V), which is a constant used to convert the measured electrical signal (voltage), the voltage was multiplied, and the displacement was obtained. From the slope in the displacement-versus-voltage graph, the piezoelectric coefficient d33 was indirectly estimated, where the *x*-axis represents the applied voltage, and the displacement of the conductive tips is represented by the *y*-axis [24,25].

Measurements were taken at multiple points along the film, yielding *d*_33_ = 1.4928 nm/V. The graphs corresponding to the different measurement points are shown in Figure 14b. This result reinforces the uniformity evident in the SEM images.

In the context of energy harvesting, a higher *d*_33_ value generally indicates greater conversion of mechanical energy into electrical energy [26]. In addition to these results, it is crucial to consider other factors, such as durability, fatigue resistance, and environmental stability. Furthermore, it is important to estimate the coefficient using an alternative method to validate the results of this study.

**Figure 15:**
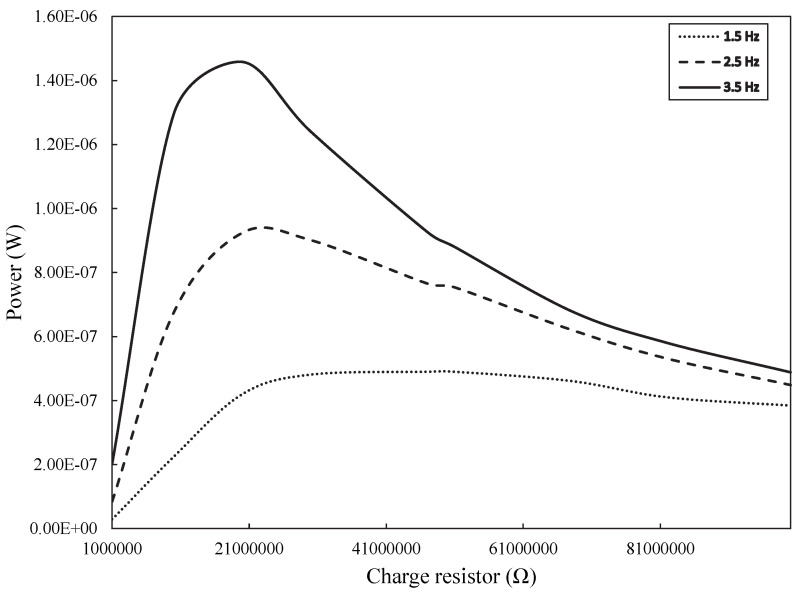
Power transfer curves for PVDF sensor.

The power (estimated by Equation (4)) for a given load resistance (*R*_x_) at each frequency is shown in Figure 15 for the PVDF sensor. At 1.5 Hz, the maximum power was 123 nW at 51 MΩ. At 2.5 Hz, it was 231 nW at 20 MΩ. At 3.5 Hz, the maximum recorded power was 365 nW at 20 MΩ. It was not possible to estimate the maximum power obtained with the CdS film because the open-circuit voltage of the film was corrupted by power line interference (60 Hz) due to the low capacitance of the film (11–16 pF). This issue could have been resolved by enclosing the sensor in a metal case (similar to a Faraday cage), but coupling the film to the system shown in Figure 2 would not be feasible. According to Equation (3), this implies that the *R*_x_ resistance to obtain maximum power must be about 10 GΩ, which is extremely high. However, to get an idea of how much power can be obtained using the CdS film, for a strain frequency of 1.5 Hz and *R*_x_ = 10 GΩ, the maximum power transferred to the load would be about 4 pW.

**Figure 16:**
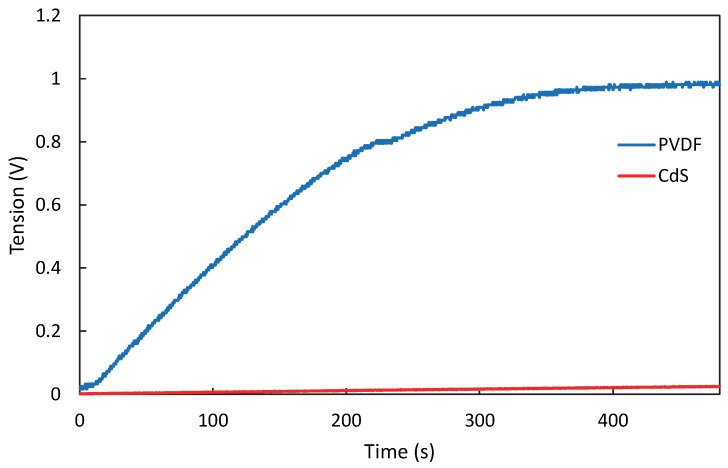
Charge curve comparison of both sensors deformed at a 3.5 Hz.

In Figure 16, the charge curves using each sensor at the higher frequency (3.5 Hz) have a difference of 956 mV. This can be attributed to the higher piezoelectric properties of PVDF and its higher capacitance, which allows for more efficient energy conversion. Although the CdS film has a high *d*_33_, the low harvested energy may be due to: (i) the electromechanical coupling factor *k_ij_*. This factor has not been estimated, since controlled tests are required to quantify the output electrical energy with respect to the input mechanical energy which is beyond the scope of this work; or (ii) impedance-matching between the piezoelectric film (CdS) and the energy conditioning circuit (LTC-3588-1, Analog Devices, Norwood, MA, USA) due to the low capacitance of the CdS film (11–16 pF). Regarding this last point, it is complicated to match the impedance between the film and the energy conditioning circuit, resulting in a lower voltage being transferred to the load.

**Figure 17:**
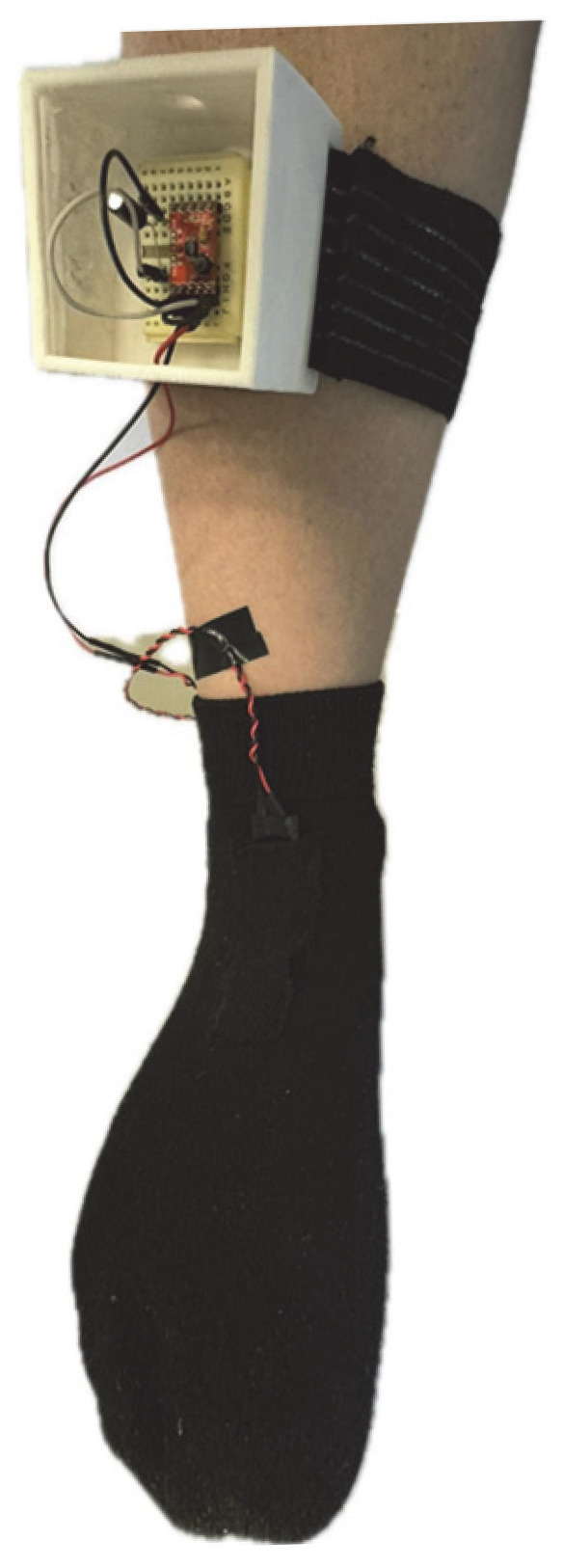
Sock with piezoelectric sensors and circuitry used to collect energy while walking.

The custom-made sock was designed to securely hold the sensors, ensuring consistent contact with the foot to maximize energy transfer (Figure 17). A flexible textile material allows adaptability to different foot sizes while maintaining sufficient compression to prevent sensor displacement during movement. The energy-conditioning circuit shown in Figure 6 was placed on the calf, secured by an elastic band, in such a way that it did not cause discomfort or pain while the subject walked.

**Figure 18:**
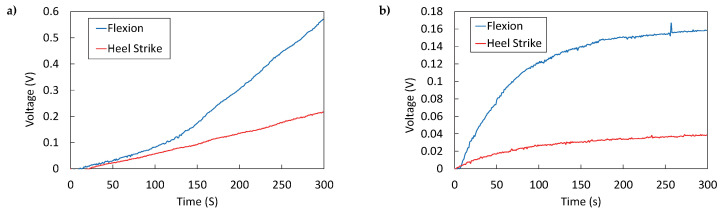
Comparison of charge curves obtained from walking: (**a**) flexion and heel strike using the PVDF sensor, and (**b**) flexion and heel strike using the CdS sensor. Volunteer 3.

**Figure 19:**
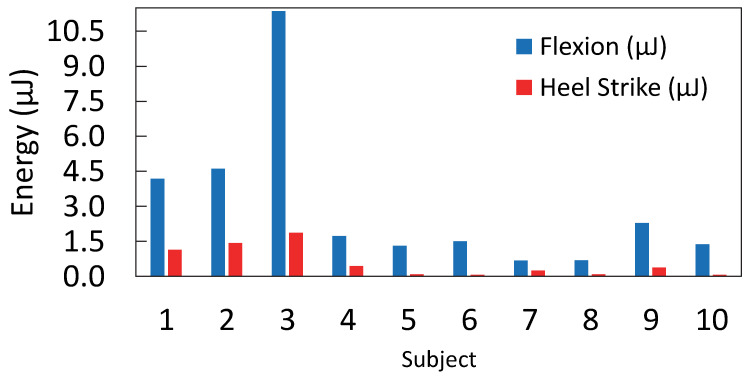
Comparison of the energy harvested from different zones on each subject using the PVDF sensor.

The PVDF sensor generated significantly more energy than the CdS sensor during walking (Figure 18a,b). This difference was more pronounced at the talocrural joint than in the plantar region. The talocrural joint yielded higher energy due to greater deformation during dorsiflexion and plantarflexion, which deforms the sensor and generates more voltage. This may be because, during walking, the foot’s impact with the ground occurs at the beginning of the stance phase, which represents 60% of the total gait cycle, of which heel strike accounts for 10%. In contrast, dorsiflexion and plantarflexion of the foot occur during 80% [27]. These results were consistent among all the volunteers, where the talocrural area exhibited greater energy generation than the plantar area of the foot (Figure 19). The differences among subjects lie in the placement of the sock on each subject’s foot. Although different sock sizes were used, each subject’s foot morphology was distinct, which affected the position of the sensors relative to the talocrural joint. Those where the sensors were centered on the talocrural joint generated the greatest amount of energy (subject 3), while those where the sensors were slightly further from the talocrural joint generated less energy (subject 7). It could be argued that there is a certain dependence between foot size and the energy generated, but this difference could also be due to the fit of the sock on the foot. In future studies it would be interesting to analyze whether there is a correlation between foot size and energy harvesting from ankle flexion during gait.

## 5. Conclusions

The CdS thin films exhibited an absorption border at 523 nm and a band gap of 2.37 eV. The deposition of the material was homogeneous, with ion-by-ion growth of the thin film and defined grain boundaries. The CdS showed a hexagonal crystalline structure, which is ideal for piezoelectric behavior due to the non-centered crystal structure. From an electrical perspective, the developed CdS film exhibited a capacitive behavior ranging from 11 pF to 16 pF. This characteristic complicates impedance-matching with power-conditioning circuits, resulting in lower voltage being transferred to the load than that obtained by the PVDF film, which had a capacitance of 2.85 nF. Regarding the piezoelectric coefficient, the *d*_33_ value was 1.4928 nm/V, indicating high efficiency of conversion from mechanical to electrical energy.

The PVDF sensor harvested more energy in the talocrural joint than in the plantar region, obtaining 578 mV when walking for 5 min. This voltage would increase if more sensors were used to collect energy from ankle flexion. This result matched with the low-frequency response characterization, which yielded a voltage of approximately 700 mV at walking-related frequencies (1.5 Hz–2.0 Hz). Similarly, for the CdS sensor, the highest energy harvested was also observed in the talocrural joint, where a charging voltage of 158 mV was measured, compared to the sinusoidal response characterization value of 226 mV.

The maximum energy harvested was 11.359 μJ in the talocrural joint and 1.865 μJ in the plantar region using the PVDF sensor. With the CdS sensor, 0.854 μJ was harvested in the talocrural joint and 0.052 μJ in the plantar region. These differences can be attributed to greater deformation in the transverse and longitudinal axes of the sensor, as well as the impact dynamics of foot contact with the ground. In the plantar region, energy harvesting is limited to heel–ground contact, producing a single impulse. In contrast, in the talocrural region, energy is harvested throughout 80% (approx.) of the gait cycle. These findings confirm that the talocrural joint provides more energy during gait, highlighting its relevance for future research in wearable energy-harvesting devices.

Future studies will focus on developing an array of piezoelectric sensors distributed across the foot region to generate sufficient energy for wearable devices that measure human physiological parameters. This approach aims to reduce reliance on environmentally harmful energy storage systems. Additionally, further exploration of CdS-based piezoelectric materials will focus on improving film thickness (≥28 μm) and optimizing their structural properties to fully exploit their piezoelectric potential.

## Data Availability

The original contributions presented in the study are included in the article, further inquiries can be directed to the corresponding author.
